# Therapeutic potential of mackerel-derived peptides and the synthetic tetrapeptide TVGF for sleep disorders in a light-induced anxiety zebrafish model

**DOI:** 10.3389/fphar.2024.1475432

**Published:** 2024-11-11

**Authors:** Yang Wang, Lei Gu, Haijing Zhang, Junbao Wang, Xichang Wang, Yu Li, Shiwei Chai, Changhua Xu

**Affiliations:** ^1^ Department of Pharmacy, First Teaching Hospital of Tianjin University of Traditional Chinese Medicine and National Clinical Research Center for Chinese Medicine Acupuncture and Moxibustion, Tianjin, China; ^2^ College of Food Science and Technology, Shanghai Ocean University, Shanghai, China; ^3^ R&D department, Shanghai Engineering Research Center of Aquatic-Product Processing & Preservation, Shanghai, China; ^4^ R&D department, Laboratory of Quality and Safety Risk Assessment for Aquatic Products on Storage and Preservation, Ministry of Agriculture, Shanghai, China; ^5^ R&D department, National R&D Branch Center for Freshwater Aquatic Products Processing Technology, Shanghai, China

**Keywords:** bioactive peptide, mackerel, sleep-promoting, γ-aminobutyric acid, insomnia, zebrafish

## Abstract

**Introduction:**

Anxiety-like insomnia is a known risk factor for the onset and worsening of certain neurological diseases, including Alzheimer’s disease. Due to the adverse effects of current anti-insomnia medications, such as drug dependence and limited safety, researchers are actively exploring natural bioactive compounds to mitigate anxiety-like insomnia with fewer side effects. Mackerel (*Pneumatophorus japonicus*), a traditional Chinese medicine, is known for its tonic effects and is commonly used to treat neurasthenia. The use of mackerel protein extract has been shown to effectively improve symptoms of light-induced anxiety-like insomnia in a zebrafish model.

**Methods:**

This study examines the effects of mackerel bone peptides (MW < 1 kDa, MBP1) and the synthetic peptide Thr-Val-Gly-Phe (TVGF) on light-induced anxiety-like insomnia in zebrafish. The evaluation is conducted through behavioral observation, biochemical marker analysis, and gene transcriptome profiling.

**Results:**

MBP1 significantly alleviated abnormal hyperactivity and restored neurotransmitter levels (dopamine and γ-aminobutyric acid) to normal. Moreover, it mitigated oxidative stress by reducing reactive oxygen species production and malonaldehyde levels, while enhancing antioxidant enzyme activities (superoxide dismutase and catalase). This was further attributed to the regulation of lipid accumulation and protein homeostasis. Furthermore, MBP1 ameliorated sleep disturbances primarily by restoring normal expression levels of genes involved in circadian rhythm (*per2* and *sik1*) and visual function (*opn1mw2*, *zgc:73075*, and *arr3b*). Molecular docking analysis indicated that TVGF exhibited good affinity for receptors linked to sleep disturbances, including IL6, HTR1A, and MAOA. TVGF exhibited sedative effects in behavioral assays, mainly mediated by regulating the normal expression of genes associated with circadian rhythm (c*ry1bb*, *cry1ba*, *per2*, *per1b* and *sik1*), visual function (*opn1mw1*, *gnb3b*, *arr3b*, *gnat2*), purine metabolism (*pnp5a*), and stress recovery (*fkbp5*).

**Discussion:**

These findings suggest that MBP1 and TVGF could be promising therapies for light-induced anxiety-like insomnia in humans, offering safer alternatives to current medications. Additionally, the regulation of genes related to circadian rhythm and visual perception may be a key mechanism by which MBP1 and TVGF effectively relieve anxiety-like insomnia.

## Graphical Abstract

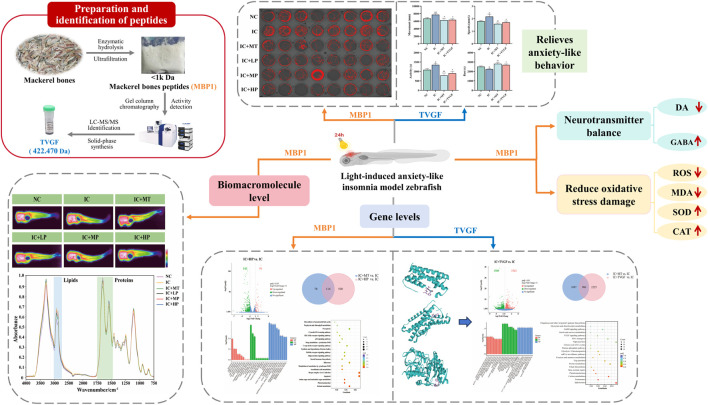

## 1 Introduction

Insomnia is a prevalent public health problem, and normal biological rhythms interact with environmental and behavioral cycles to balance nocturnal sleep homeostasis and moderate emotional regulation (Baglioni et al., 2010). However, in the technology-driven industrial society, this coordination is often disturbed by contemporary changes in living environments, light exposure patterns, work and social schedules, and underlying biological factors (Fuchs et al., 2023). Light plays an essential role in regulating human sleep-wake cycles, with the main mechanism involving the suprachiasmatic nucleus and pineal melatonin secretion controlling circadian rhythms (Meyer et al., 2022). Equally, aberrant light exposure disrupts normal pineal regulation, visual behavior, emotional homeostasis and oxidative stress (Bilotta, 2000; Ben-Moshe et al., 2014; Alifu et al., 2021). Specifically, the biological rhythm system can be suppressed by behavioral and environmental factors, causing stressful emotions such as irritability, anxiety and depression, further exacerbating the occurrence of anxiety-like insomnia (Kennair et al., 2022). This study investigates potential sleep-promoting bioactivities using the zebrafish model of light-induced anxiety-like insomnia.

Currently, drug treatments for insomnia, such as benzodiazepines, antidepressants and melatonin receptor agonists are readily prescribed. However, long-term use of these drugs is associated with potential drawbacks including high addiction, withdrawal symptoms and safety security (Morin and Benca, 2012). Potential physiological functions and mechanisms of marine-derived bioactive peptides derived from novel food and pharmaceutical resources to prevent and treat insomnia have garnered widespread scientific interest (Sridhar et al., 2021). Studies have demonstrated oyster peptides have been shown to promote sleep by increasing the levels of inhibitory neurotransmitters (5-hydroxytryptamine, γ-aminobutyric acid) and decreasing the expression of excitatory neurotransmitters (dopamine, norepinephrine), thus prolonging sleep duration in mice (Zhang et al., 2021). *Decapterus maruadsi* peptide has been shown to reverse cognitive deficits caused by sleep deprivation by alleviating oxidative stress (Zhang et al., 2019). Casein trypsin hydrolysates have been shown to relieve stress and promote sleep (Qian et al., 2021). Furthermore, a new venom peptide from the marine snail Conus *araneosu*s has been characterized as possessing sleep-inducing properties in mice (Franklin and Rajesh, 2015). These findings collectively suggest that peptides possess great potential as medical treatments to improve sleep quality.

Mackerel belongs to the Perciformes, a type of warm water pelagic fish with rich nutrition and high economic value. It has the effect of strengthening with tonics and is mainly used for chronic gastrointestinal diseases and neurasthenia (Hashimoto et al., 2017). In traditional Chinese medicine, mackerel is used for its calming and restorative properties, often processed by drying and grinding into powder or boiling with herbs to make decoctions or infusions. However, the amount of fish waste by-products around the world is increasing rapidly, encompassing fish heads, skins, viscera, scales, bones, and any remaining flesh. Utilizing these by-products is an excellent cost-recovery strategy for enhancing their commercial value and preventing environmental pollution (Nurilmala et al., 2022). To maximize the value of fish waste, this study prepared bioactive peptides from mackerel bones. Although it has been widely reported that mackerel contains biological activities such as sleep promotion, fatigue resistance, antioxidant, and iron enhancer (Wang et al., 2014; Wang et al., 2018; Wang et al., 2023; Tan et al., 2023), the effect and mechanism of derived peptides in alleviating anxiety-like insomnia remains unclear.

Therefore, this study aims to investigate the effects of mackerel bone peptides (molecular weight < 1 k Da, MBP1) on improving anxiety-like insomnia and the under-lying mechanisms using zebrafish models. A novel peptide, Thr-Val-Gly-Phe (TVGF), was identified from MBP1 through a targeted isolation method and its sedative and hypnotic properties, along with its underlying mechanisms were preliminarily explored. The findings will provide new theoretical foundations and therapeutic targets to alleviate anxiety-like insomnia and reveal the potential mechanism of sleep promotion.

## 2 Materials and methods

### 2.1 Materials and reagents

Fresh mackerel bones were supplied by Zhejiang Industrial Group Co. Ltd. (Zhoushan, Zhejiang, China). The head and remaining meat were removed, and the bones were frozen at −20°C for further processing. Melatonin (MT) was purchased from Gaoxin Chemical Glass Co., Ltd. (Shanghai, China). Zebrafish dopamine (DA) and γ-aminobutyric acid (GABA) ELISA kits were purchased from Mreda Technology Co., Ltd. (Beijing, China). 2′, 7′-Dichlorofluorescein diacetate (DCFH-DA) was obtained from Solarbio Science & Technology Co., Ltd. (Beijing, China). Superoxide dismutase (SOD), catalase (CAT), and lipid peroxidation (MDA) detection kits were purchased from Yuanye Bio-Technology Co., Ltd. (Shanghai, China). TVGF and TVGF-Acp-FITC were synthesized by GL Biochem (Shanghai, China) Ltd. All other chemicals and reagents utilized in this study were purchased from local commercial establishments and were analytical grade.

### 2.2 Zebrafish maintenance and exposure protocols

Wild-type adult zebrafish (AB strain) were obtained from the Key Laboratory of Exploration and Utilization of Aquatic Genetic Resources, Ministry of Education, Shanghai Ocean University (Shanghai, China). All animal experiments were based on the institutional ethical guidelines of the Shanghai Ocean University Experimentation Ethics Review Committee (SHOU-DW-2016-002). Zebrafish were maintained under standard environmental conditions: 28°C ± 0.5°C, a 14/10-h light/dark cycle, and a pH of 6.8–7.2. The night before spawning, adult zebrafish were placed in the mating tank overnight (male: female = 1: 1), with baffles separating males and females. The baffles were re-moved the next morning, allowing natural mating. Embryos were collected and incubated at 28°C ± 0.5°C in a thermostat incubator until used for exposure experiments. During this period, maintain fresh water daily and promptly remove dead embryos and debris.

At 96 h post-fertilization (hpf), zebrafish larvae were exposed to 200 lux of white light continuously for 24 h to establish a model of anxiety-related insomnia. Subsequently, larvae were randomly assigned to groups and treated with melatonin, MBP1, and TVGF for 24 h prior to transfer to 96-well plates. Including the normal control group (NC) without 24-h light exposure; anxiety-like insomnia model zebrafish control group (IC); IC larvae treated with 10^−3^ mol/L melatonin (IC + MT); IC larvae treated with low (0.03 mg/mL), medium (0.05 mg/mL), and high (0.3 mg/mL) dose of MBP1 (IC + LP, IC + MP, IC + HP); IC larvae treated with 0.2 mg/mL TVGF (IC + TVGF). All experiments were performed in triplicate and 50% of the solution in each dish was re-placed with fresh solution every 24 h during the exposure period.

### 2.3 Preparation of MBP1

Based on our previous research methodology, mackerel bones were initially defatted, chopped, and mixed with distilled water (water-to-bone ratio = 3.62:1). Papain and trypsin (1:1 enzyme ratio) were then added for hydrolysis, with a total enzyme concentration of 5,000 U/g. Hydrolysis was carried out at 62°C and pH 7.0 for 4 h under water bath oscillation (120 rpm). Following enzymatic hydrolysis, the supernatant was obtained through enzyme inactivation, centrifugation, 1% activated carbon decolorization, and filtration. MBP1 was separated using an ultrafiltration membrane with a molecular weight cutoff of less than 1 kDa (Sigma-Aldrich, USA) and lyophilized to obtain MBP1 powder (The molecular weight distribution analysis has been conducted to assess purity.) for subsequent experiments.

### 2.4 Locomotor behavioral analysis in zebrafish

Zebrafish at 120 hpf were rinsed three times with culture water and individually transferred to 48-well plates (1 larva/well, n = 8/group). Prior to formal testing, larvae were gently placed in Daniovision (Noldus, Netherlands) under dark environment for 10 min to acclimate. Locomotor behavior was monitored and recorded for 1 h using the Daniovision system, with a resolution of 1,024 × 768 pixels and a frame rate of 25 frames per second (fps). Subsequently, total moving distance, swimming speed, active time and rest time were analyzed using Ethovision XT11 software.

### 2.5 Determination of neurotransmitter content

Following exposure experiments, zebrafish (n = 60/group) at 120 hpf were collect-ed and homogenized in an ice bath. The homogenate was then centrifuged at 3,000–4,000 rpm for 15 min at 4°C to obtain the supernatant. DA and GABA concentrations in zebrafish were quantified using commercially available ELISA kits. The concentrations of DA and GABA in the supernatant were determined according to the manufacturer’s instructions.

### 2.6 Evaluation of oxidative stress and related enzyme activities of MBP1

The levels of reactive oxygen species (ROS), malondialdehyde (MDA) concentration and antioxidant enzyme activity were measured to evaluate the experimental induced oxidative stress response of zebrafish (Guru et al., 2021). Larvae at 120 hpf after exposure were randomly transferred to 48-well plates and incubated with 20 μg/mL DCFH-DA for 1 h at 28.5°C in the dark for staining. Stained larvae were then washed three times with PBS buffer and anesthetized with 0.04% tricaine solution. Later, zebrafish were imaged in the lateral position under a fluorescence microscope (OLYMPUS IX71, Japan), and the fluorescence intensity of the zebrafish (n = 5/group) was quantified by ImageJ software to estimate the level of ROS production. The larvae (n = 120/group) at 120 hpf after exposure were collected and rinsed three times with PBS buffer, then homogenized in an ice bath as required and centrifuged (10,000–12,000 rpm, 15–20 min, 4°C) to collect the supernatant. Later, the supernatant was used to determine the levels of MDA, SOD and CAT according to the manufacturer’s instructions.

### 2.7 Zebrafish infrared transmission imaging spectral data acquisition

Zebrafish from each experimental group were randomly selected and fixed in the lateral position using OCT freezing embedding agent (n = 3/group). Tissue sections of 5 μm thickness were cut with a frozen slicer (Leica CM 1950, Germany) and adhered to the ZnSe windows. The windows were then dried in a desiccator (40°C, 12 h) to eliminate moisture interference for IR imaging. Subsequently, optical imaging image and infrared spectral data of the zebrafish cross-section were acquired using a Spotlight 400 system (PerkinElmer, United States). Spectral acquisition parameters were as follows: wavenumber ranged from 4,000 to 750 cm^−1^, resolution was 4 cm^−1^, pixel size was 6.25 μm, and each pixel was scanned 16 times. PerkinElmer spectral software (Version 10.6.4) was used to extract spectral information from the head, abdomen, and tail of the zebrafish (five for each part) randomly and evenly from the obtained chemical imaging map for processing and analysis. Finally, PeakFit software (Version 4.12) was employed to analyze the percentage of secondary structures based on the extracted spectral data.

### 2.8 Transcriptomics

After the exposure experiment, zebrafish (n = 20/group) were frozen with liquid nitrogen and shipped to Novogene Biotech Co., Ltd. (Beijing, China) for transcriptome sequencing analysis based on the Illumina sequencing platform. In brief, total RNA was extracted from zebrafish using the TRIzol kit (Thermo Fisher Scientific, USA), and the integrity and quality of total RNA were accurately detected and strictly controlled using the Agilent 2100 bioanalyzer. Qualified RNA samples were subjected to Illumina Hi SeqTM sequencing after mRNA enrichment, double-stranded cDNA synthesis, end repair, poly-A enrichment, fragments selection, PCR, and library quality inspection. In order to obtain clean reads, the quality and reliability of data analysis can be ensured by removing reads containing sequencing adapters, removing reads with unknown base, and removing low-quality reads (the number of bases with Q phred ≤20 bases for more than 50% of the whole read length). Later, the clean reads were aligned to the reference genome using Hisat2 v2.0.5 (the reference genome and gene model annotation files were downloaded from the genome website). Deseq2R software was used to analyze the differential expression of zebrafish in different groups and modify the *p*-value obtained by the original hypothesis test (padj <0.05, |log2foldchange| >1). Finally, in order to further explore the differences in functions and pathways of DEGs, used cluster Profiler 3.8.1 software for GO and KEGG enrichment analysis of DEGs.

### 2.9 Identification and synthesis of sleep-promoting peptides from MBP1

The protocol for the isolation, purification and identification of peptides with hypnotic activity of MBP1 was based on our previous research (Wang et al., 2014). Briefly, the MBP1 solution (8 mg/mL) was purified by chromatography column (1.6 × 60 cm) equipped with Sephadex G-15. Ultimately, three fractions were obtained, lyophilized and their sleep-promoting activities were investigated by the light-induced sleep disturbances zebrafish model. LC-MS/MS technology was used to identify the peptide sequence by comparing and matching the database. The dominant peptide sequence TVGF was identified by mass spectrometry and synthesized by solid-phase synthesis technology. The purity and molecular weight of TVGF were determined by ONO LC-2000 (4.6 mm × 250 mm, 5 μm) HPLC and mass spectrometry (Agilent-6125B).

### 2.10 Molecular docking

The 3D structure of TVGF was constructed using PyMOL software and processed with AutoDock4 software, such as hydrogenylation, charge balance, and then saved as pdbqt files. The PDB ID of the relevant receptor proteins was obtained from PDB (https://www.rcsb.org/) and then optimized by PyMOL software, including dehydration, hydrogenation, and deligandization. Autodock 4 software was used to perform semi-flexible docking of the receptor and ligand. The operation steps included setting the GridBox size, running AutoGrid molecular docking, observing the molecular con-formation of the docking and analyzing the docking data.

### 2.11 Statistical analysis

All experimental studies were expressed as mean ± standard deviation. The data were analyzed by ANOVA test (SPSS 21.0 statistical software). Tukey test was used to analyze the significant difference between the mean values of each parameter. Relevant data charts were generated using GraphPad Prism 8.0.2 and Microsoft Excel 2019.

## 3 Results

### 3.1 Effects of MBP1 on zebrafish behavior and neurotransmitter levels

**FIGURE 1:**
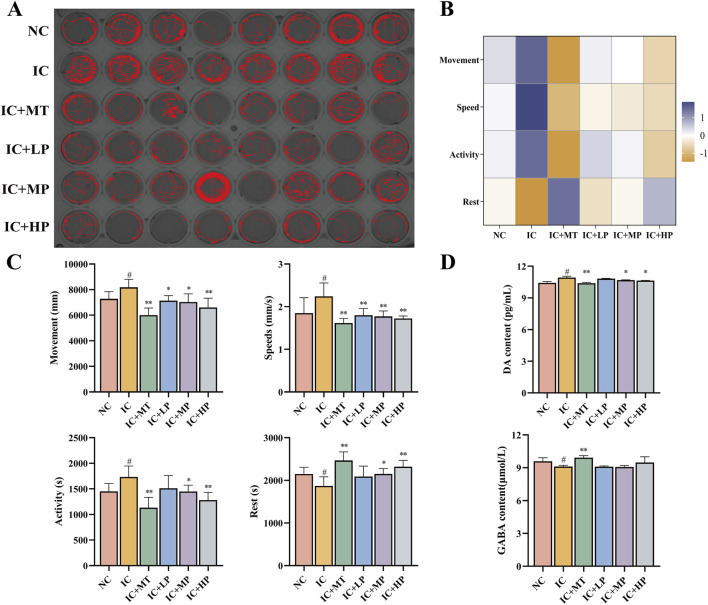
Effects of MBP1 on locomotor behavior and neurotransmitter levels in zebrafish with anxiety-like insomnia. **(A)** Experimental digital track map. The red lines are the motion tracks (n = 8 in each group). **(B)** Heatmap of behavioral indicators analysis. **(C)** Changes in behavioral indices of zebrafish treated with MT and different doses of MBP1. **(D)** Changes in neurotransmitter levels (DA and GABA) in zebrafish (n = 60 in each group). Student’s t-test, #*p* < 0.05 vs. the NC group; *p < 0.05 and **p < 0.01 vs. the IC group.

Long-term light exposure can disrupt the circadian rhythm of zebrafish and make them extremely excited and active. Therefore, the impact of long-term light exposure on the locomotor behavior of zebrafish can be examined by tracking locomotor trajectories (Figure 1A) (Gong et al., 2017). The IC group exhibited more intricate and disordered movement trajectories compared to the NC group, characterized by increased movement distance, swimming speed, active time, and reduced rest time (Figures 1B,C). Treatment with MBP1 at varying concentrations effectively mitigated these behavioral changes, reducing locomotor activity and promoting rest time. The highest dose of MBP1 (IC + HP) yielded the most pronounced improvement, approaching the control group and exhibiting comparable effects to the melatonin-treated group (IC + MT). Thus, it can be preliminarily determined that the anxiety-like movement of zebrafish is alleviated under the protection of MBP1 active peptide.

Neurotransmitter imbalances are crucial factors in the development of sleep disturbances. DA in sleep-wake regulation is considered as a wakefulness-promoting ex-citatory neurotransmitter (Herrera-Solis et al., 2017), while GABA is a crucial inhibitory neurotransmitter for the initiation and maintenance of sleep in the brain (Morgan et al., 2012). As depicted in Figure 1D, exposure to prolonged light caused an imbalance in the neurotransmitter system, leading to increased DA and decreased GABA concentrations in zebrafish, ultimately causing hyperkinesis and resulting in insomnia. The effect of the IC + HP group showed a significant reversal advantage in light-induced anxiety-like insomnia, and the effect was close to that of the IC + MT group, while the effect of the IC + LP and IC + MP groups was relatively slight (Figure 1D). At higher doses of MBP1, the effect on neurotransmitter regulation was comparable to that of the positive drug group. Additionally, the changes in neurotransmitter levels in all treatment groups remained within a safe range, ensuring no other physiological damage was caused. Therefore, MBP1 can act with the neurotransmitter system to improve anxiety-related activities.

### 3.2 Effect of MBP1 on oxidative stress in zebrafish

**FIGURE 2:**
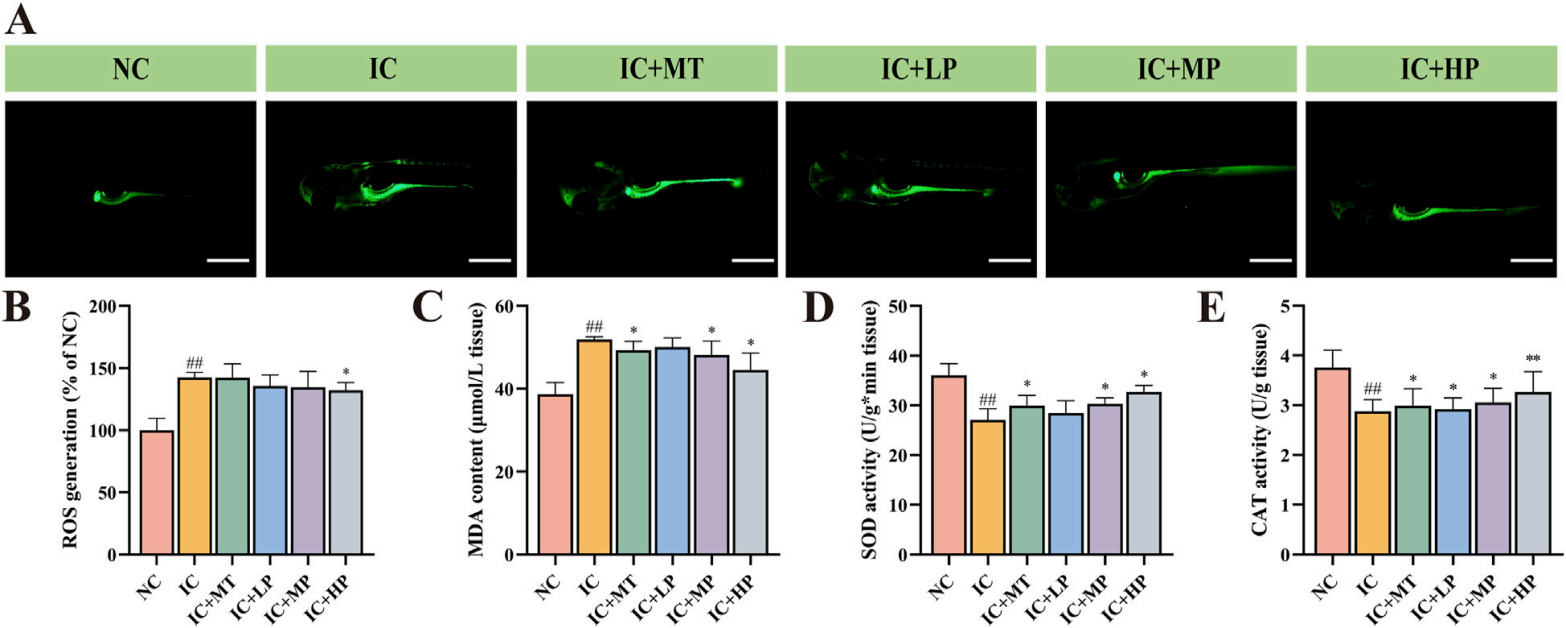
Effects of MBP1 on oxidative stress in zebrafish with anxiety-like insomnia. **(A)** Representative fluorescence images of each group stained with 2′,7′-dichlorofluorescein diacetate (DCFH-DA) to visualize reactive oxygen species (ROS) (n = 5 in each group). Scale bar, 500μm; **(B)** ROS accumulation levels (n = 120 in each group). Data are expressed as percentage of NC group; **(C)** Malondialdehyde (MDA) content (n = 120 in each group); **(D)** Superoxide dismutase (SOD) activity (n = 120 in each group); **(E)** Catalase (CAT) activity (n = 120 in each group). Student’s t-test, #*p* < 0.05 vs. the NC group; *p < 0.05 and **p < 0.01 vs. the IC group.

As depicted in Figures 2A–C, compared to the NC group (ROS, 100.00%; MDA, 38.68 μmol/g), the IC group exhibited significantly increased levels of ROS (142.43%) and MDA (51.88 μmol/g), visualized by enhanced green fluorescence around the yolk sac, head, and eyes. Treatment with various concentrations of MBP1 effectively attenuated these effects, reducing ROS production and MDA levels in a dose-dependent manner. Among them, the IC + HP group (ROS, 132.07%; MDA, 44.54 μmol/g) demonstrated the most significant improvement, focusing the fluorescence distribution primarily to the yolk sac, indicating its potential to regulate oxidative stress. In addition, compared with NC group (SOD, 36.02 U/g; CAT, 3.76 U/g), the activities of SOD (27.12 U/g) and CAT (2.87 U/g) in IC group were significantly reduced in the IC group. However, treatment with various doses of MBP1 reversed this trend, enhancing the expression levels of endogenous antioxidant enzymes (SOD, CAT) in a dose-dependent manner (Figures 2D,E). These findings indicate that MBP1 can alleviate anxiety-like insomnia through enhancing the activity of the antioxidant defense system.

### 3.3 Impact of MBP1 on zebrafish chemical composition distribution

**FIGURE 3:**
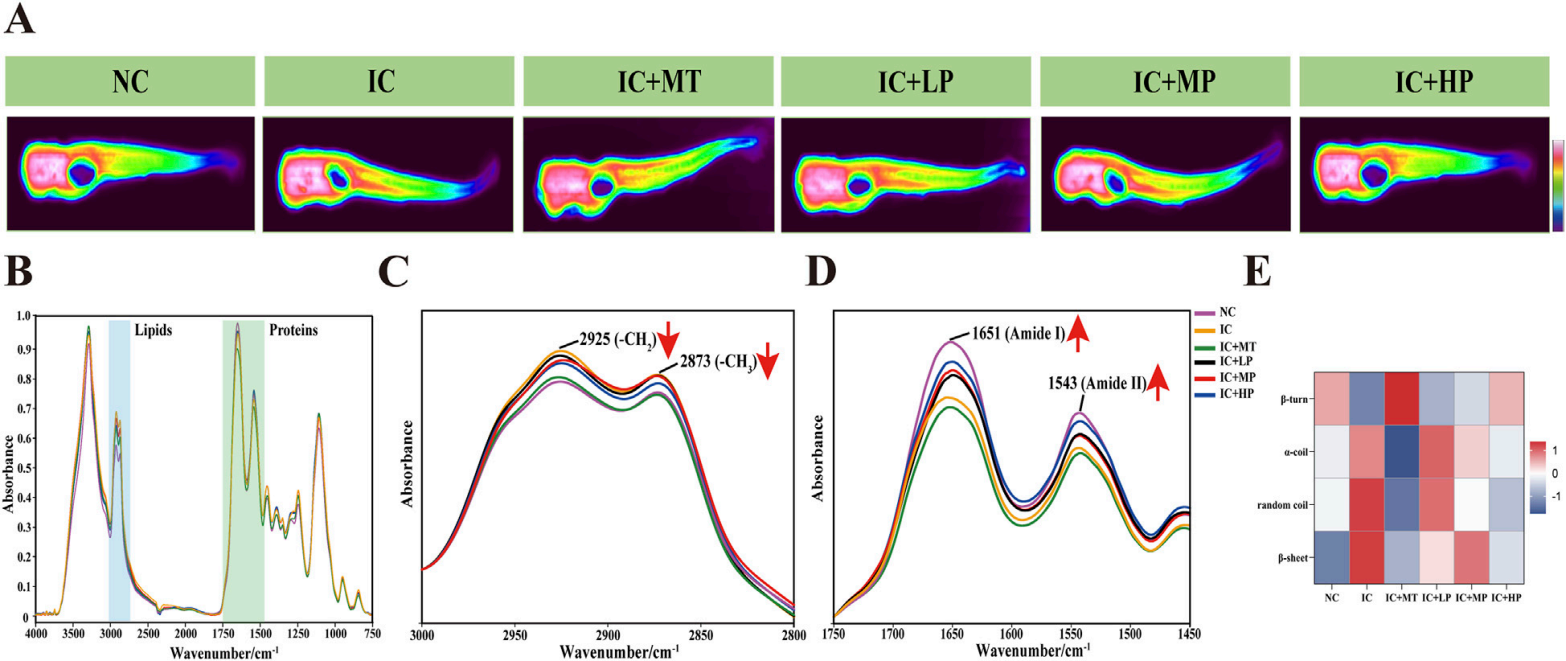
Effects of different doses of MBP1 on chemical composition changes in zebrafish with anxiety-like insomnia. **(A)** Representative infrared transmission images of each group (n = 3 in each group); **(B)** The integrated infrared average spectra (4,000–750 cm^−1^) were extracted from the head, abdomen, and tail of zebrafish based on infrared transmission imaging. **(C)** IR Spectra of zebrafish in the lipid band (3,000–2,800 cm^−1^). **(D)** IR Spectra of zebrafish at protein band (1750–1,450 cm^−1^). **(E)** Heat map of zebrafish protein secondary structure in different treatment groups.

Infrared (IR) transmission spectroscopy can simultaneously collect hundreds of pixel spectra with a high signal-to-noise ratio and low distortion, demonstrating the clear correlation between molecular structure and spectral characteristics (Chen et al., 2013). It can intuitively and completely analyze the spatial distribution and chemical composition information of biomacromolecules such as lipids and proteins in the microscopic region of biological tissues (Table 1) (Hou et al., 2020). It is observed from the IR transmission images of zebrafish cross-section that a substantial presence of proteins (red areas) occupies a significant proportion of the entire cross section (Figure 3A). Analysis of IR spectra (Figure 3B) extracted from IR transmission images revealed alterations in chemical composition following treatment with different doses of MBP1. Insufficient sleep and circadian rhythm disorder increase the risk of obesity and central obesity (Cai et al., 2018), and there is a latent correlation between lipid accumulation and tau aggregate formation (Zhao et al., 2023). In line with these findings, the characteristic lipid peaks at 2,925 cm^−1^ (υ_as_ (CH2)) and 2,873 cm^−1^ (υ_s_ (CH3)) were significantly elevated in the IC group compared to the NC group (Figure 3C), indicating increased lipid content in zebrafish experiencing sleep deprivation. Treatment with varying doses of MBP1 effectively reduced these lipid peaks in a dose-dependent manner, gradually approaching the NC group levels (Figure 3C), demonstrating its potential to mitigate lipid accumulation associated with insomnia.

**TABLE 1:** Peak assignments of the infrared spectrum of zebrafish.

Wavenumber (cm^-1^)	Assignment
3,000–2,800	Lipid (υ_as_ (CH_3_), υ_as_ (CH_2_), υ_s_ (CH_3_), υ_s_ (CH_2_))
1,600–1700	Protein (Amide-I)
1,580–1,500	Protein (Amide-II)
1,640–1,620	Protein (β-sheet)
1,650–1,640	Protein (random coil)
1,658–1,650	Protein (α-helix)
1,660–1,695	Protein (β-turn)

*Note: υ, stretching vibration; δ, bending vibration; s, symmetry; as, antisymmetric.

Similarly, the characteristic peaks associated with Amide I and Amide II, indicative of protein structure, were significantly lower in the IC group compared to the NC group. However, MBP1 treatment led to a gradual restoration of these peaks towards NC levels with increasing MBP1 concentration. This trend was not observed in the melatonin (MT) treated group (Figure 3D). Further analysis of the protein region using second derivative infrared spectroscopy (SD-IR) revealed alterations in protein secondary structure within the Amide I band (Figure 3E). Compared to the NC group, the IC group exhibited increased α-helix, random coil, and β-sheet content while β-turn content decreased. This shift in protein secondary structure is consistent with the hypothesis that excessive ROS generation during sleep deprivation leads to misfolded proteins and protein aggregation (Vitaterna et al., 2019). Interestingly, the secondary structures of proteins in the IC + LP, IC + MP, and IC + HP groups were also interconverted, exhibiting a trend of α-helix, random coil, and β-sheet content decreased, β-turn content increased and gradually approached a normal level, which was particularly obvious in the IC + HP group. These results indicated that MBP1 may prevent the accumulation of excessive β-sheet protein to a certain extent, thereby preventing the aggregation of tau protein and mitigating the adverse effects of light-induced sleep disturbances on the imbalance of protein homeostasis.

### 3.4 Transcriptome analysis of zebrafish by MBP1

**FIGURE 4:**
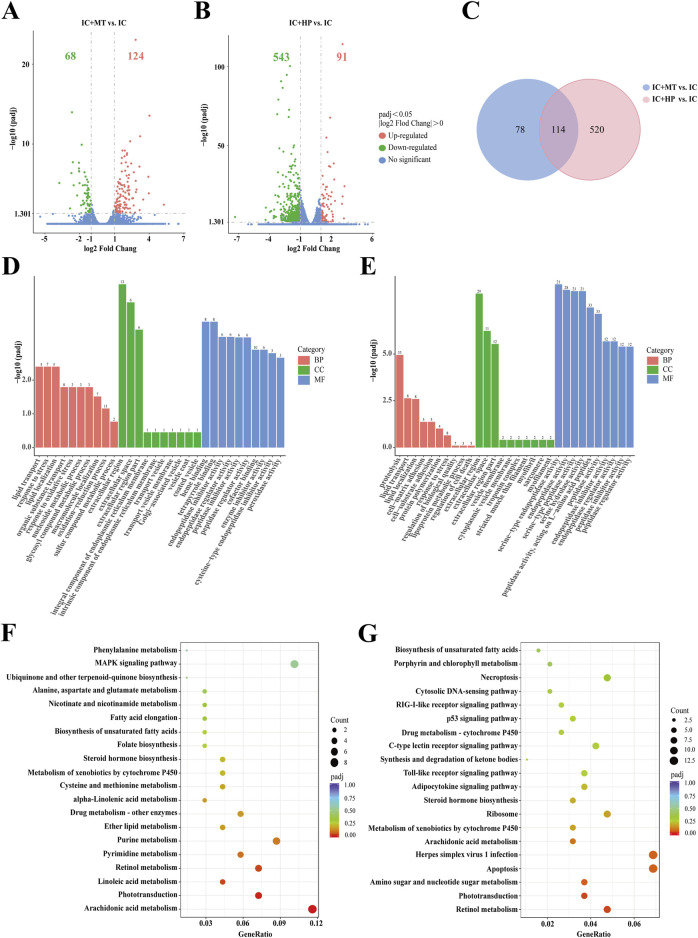
Transcriptome sequencing analysis of MBP1 treated zebrafish. Volcano plots depicting differentially expressed genes (DEGs) were generated for both the IC + MT group **(A)** and IC + HP group **(B)** groups, compared to the IC group (n = 20 in each group). **(C)** Venn diagrams illustrated the overlap and unique DEGs identified in the two comparisons: IC + MT vs. IC and IC + HP vs. IC. The bar plots of GO enrichment pathways in the IC + MT group **(D)** and IC + HP group **(E)** compared with the IC group indicated the top 30 pathways of different genes. The scatter plots of KEGG enrichment pathways in the IC + MT group **(F)** and IC + HP group **(G)** compared to the IC group reveal the top 20 pathways based on different genes.

RNA-Seq analysis was performed on zebrafish in the IC, IC + MT and IC + HP groups. Following quality control analysis, adapters and low-quality sequences were removed. Clean Q30 and Q20 results indicated the transcriptome sequencing was of high quality. The biological replicates of zebrafish in each group were highly correlated, and the results of subsequent transcriptome analysis were accurate and credible (Supplementary Tables S1 and S2). Differentially expressed genes (DEGs) were identified based on the criteria of |log2 FC| > 0 & padj <0.05. Volcano plot analysis revealed 192 DEGs (68 downregulated, 124 upregulated) between the IC + MT and IC groups, and 634 DEGs (543 downregulated, 91 upregulated) between the IC + HP and IC groups (Figures 4A,B). There were 114 common DEGs in the two comparison combinations, while 78 and 520 DEGs were uniquely regulated by the IC + MT and IC + HP groups, respectively (Figure 4C). To further explore the potential mechanisms by which MBP1 ameliorates light-induced sleep disturbances, |log2 FC| > 1 & padj <0.05 were used as the default criteria for difference significance, and 15 DEGs with greater significance in the two comparison combinations were selected for comparative analysis (Table 2).

**TABLE 2:** Comparison of the top 15 DEGs in the IC + MT and IC + HP groups compared with IC group.

Gene name	IC + MT vs. IC		IC + HP vs. IC	
	log2 FC	Padj	log2 FC	Padj
*per2*	+2.84	8.97 × 10^−24^	+3.10	8.22 × 10^−115^
*rps29*			−2.02	6.48 × 10^−101^
*zgc:111983*			−2.33	2.39 × 10^−95^
*si:ch211-117m20.5*			−2.88	2.63 × 10^−91^
*zgc:100868*			−2.73	3.63 × 10^−87^
*si:dkey-239b22.1*			−3.14	1.03 × 10^−79^
*sec23b*			−2.10	2.97 × 10^−73^
*opn1mw2*	−1.82	1.21 × 10^−10^	−3.25	1.84 × 10^−70^
*AC024175.18*			−2.13	1.05 × 10^−68^
*zgc:73075*	+1.69	6.50 × 10^−10^	+1.89	2.96 × 10^−68^
*arr3b*			+1.53	1.11 × 10^−55^
*rpl38*			−1.62	9.81 × 10^−51^
*sik1*			−1.68	1.89 × 10^−49^
*si: dkey-17e16.17*	−2.66	1.04 × 10^−14^	−3.05	7.50 × 10^−49^
*si:ch211-207n23.2*			−2.65	9.23 × 10^−47^
*si:ch211-161h7.5*	+4.03	2.78 × 10^−14^		
*cry5*	+3.23	1.07 × 10^−11^		
*si:ch211-170n20.3*	+2.36	4.55 × 10^−11^		
*soul5*	+1.90	6.50 × 10^−10^		
*agxtb*	+2.09	1.41 × 10^−9^		
*tfa*	+2.08	2.33 × 10^−9^		
*cry-dash*	+1.88	3.53 × 10^−9^		
*zgc:153154*	+1.65	5.57 × 10^−9^		
*elovl7b*	−2.59	1.93 × 10^−8^		
*ddb2*	+1.99	2.04 × 10^−8^		
*si: busm1-234g15.3*	−2.06	2.26 × 10^−8^		

*Note: log2 FC: log2 Fold Change; −: gene downregulation; +: gene upregulation.

The results indicate that the core circadian clock gene per2 was upregulated in both the IC + MT and IC + HP groups compared to the IC group, suggesting that it contributed to the restoration of circadian rhythm function (Vitaterna et al., 2019). The genes involved in visual perception and retinal development, *opn1mw2* was downregulated and *zgc:73075* was upregulated (Sun et al., 2018). *si:dkey-17e16.17* gene, which is involved in the Golgi transporter system was downregulated. Additionally, the IC + HP group could individually upregulate the visual perception and retinal light sensitivity gene *arr3b* (Renninger et al., 2011), and the gene *sik1* regulating circadian rhythm behavior and energy metabolism was downregulated (Jagannath et al., 2013). These transcriptional changes are consistent with previous studies demonstrating the influence of circadian rhythm networks on visual function and the presence of circadian rhythms in various visual behaviors in zebrafish (Shi et al., 2019).

Gene Ontology (GO) and Kyoto Encyclopedia of Genes and Genomes (KEGG) enrichment analyses were subsequently performed to explore the functional characteristics of these DEGs. Comparison of the IC and IC + MT groups revealed significant enrichment of GO terms related to lipid transport and response to stress in biological processes, while the extracellular region was the most enriched cellular component. Heme binding was the predominant molecular function enriched in this comparison (Figure 4D). GO enrichment analysis revealed that the IC + HP group was significantly enriched in proteolysis and lipid transport as compared to the IC group, corroborating the findings from the multi-molecule spectroscopy analysis. This was probably at-tributed to aberrant expression of circadian genes induced by insomnia, leading to overproduction and overaccumulation of clock proteins. The process involved selectively degrading clock proteins to regulate circadian rhythms (Tamaru and Takamatsu, 2018). The extracellular region was the most enriched cellular component in this comparison, and serine-type endopeptidase activity was the predominant molecular function (Figure 4E). The selected DEGs were subjected to KEGG analysis and enriched into typical signaling pathways (Figures 4F,G). The results revealed that the IC + MT group significantly enriched KEGG pathways related to arachidonic acid metabolism, phototransduction, and linoleic acid metabolism. Similarly, IC + HP group enriched pathways related to retinol metabolism, phototransduction, and amino sugar and nucleotide sugar metabolism.

### 3.5 Molecular docking analysis of TVGF interactions with sleep-related receptor proteins

**FIGURE 5:**
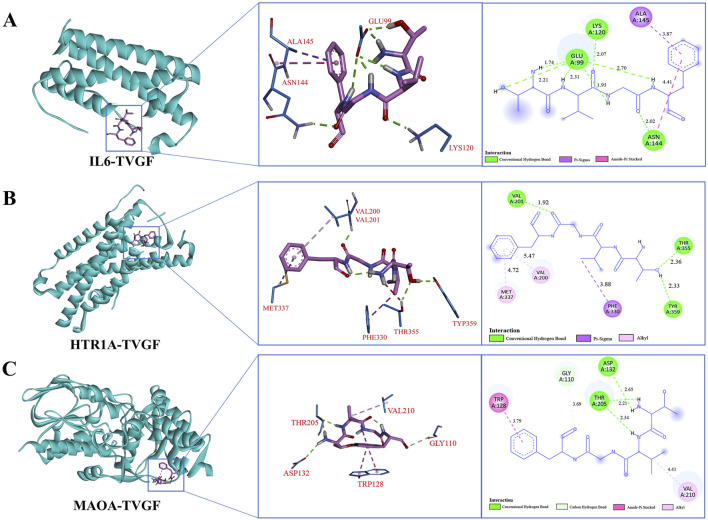
Molecular docking model of TVGF with receptor proteins associated with anxiety-like insomnia. The 3D and 2D molecular interactions of **(A)** IL6, **(B)** HTR1A, and **(C)** MAOA with the active sites of TVGF.

According to LC-MS/MS identification results (Supplementary Table S3), the purity of TVGF (retention time 10.130 min, MW 422.470 Da) synthesized by solid-phase synthesis was over 98% based on HPLC and mass spectrometry analysis (Supplementary Figure S1). The binding ability of TVGF to sleep-related receptor proteins was further assessed using molecular docking binding energies (Table 3). Top-ranked IL6, HTR1A, and MAOA were selected as key targets to study the receptor-ligand interaction pattern (Figure 5). The occurrence of insomnia is associated with the change of the level of oxidative stress and inflammation *in vivo*, and IL6 as typical inflammatory factors with TVGF good binding energy (−6.09 kcal/mol). Residues Glu99, Lys120 and Asn144 of IL6 formed three conventional hydrogen bonds with TVGF, and Ala145 and Asn144 was bound to TVGF by Pi-Sigma and Amide-Pi Stacked, respectively (Figure 5A). HTR1A is a G-protein-coupled receptor for 5-HT that plays a role in regulating DA and 5-HT levels in the brain, thereby affecting neural activity, mood, and behavior (Xie et al., 2019). Figure 5B shows that residues Val201, Thr355 and Tyr359 of HTR1A formed three conventional hydrogen bonds with TVGF. Simultaneously, there existed alkyl interactions between TVGF and HTR1A (Val200 and Met337). Insomnia is closely related to depression, and MAOA is a key enzyme involved in the degradation of monoamine neurotransmitters and plays an important role in depression (Wang et al., 2017). As shown in Figure 5C, residues Thr205 and Asp132 of MAOA formed conventional hydrogen bonds with TVGF, while Gly110 formed carbon hydrogen bonds. Additionally, TVGF formed Amide-Pi stacked with residue TRP128 and an alkyl interaction with Val210 of MAOA. Based on these results, it could be indicated that TVGF is a potent inhibitor of anxiety-like insomnia.

**TABLE 3:** Docking affinity of TVGF with sleep-related receptor proteins.

Receptor proteins	PDB ID	Binding energy (kcal/mol)
IL-6	1ALU	−6.09
HTR1A	4IAR	−5.67
MAOA	2BXR	−5.60
DRD2	6LUQ	−4.41
AKT1	4GV1	−4.03
SLC6A4	6W2C	−3.57

### 3.6 Evaluation of TVGF to alleviate light-induced sleep disturbances behavior

**FIGURE 6:**
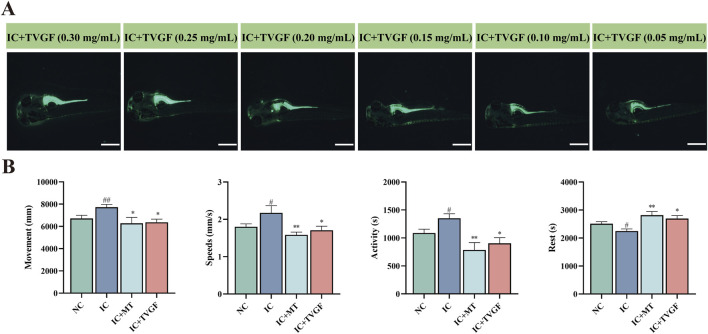
Effects of TVGF on locomotor behavior in zebrafish with anxiety-like insomnia. **(A)** Fluorescence distribution of TVGF-Acp-FITC in zebrafish (n = 3 in each group). Scale bar: 500 μm. **(B)** Changes in behavioral indicators of zebrafish after TVGF treatment (n = 8 in each group).

This study investigated the ability of TVGF to mitigate anxiety-like insomnia in zebrafish. To track the distribution of TVGF in zebrafish, a fluorescently labeled analog TVGF-Acp-FITC was synthesized, which emits green fluorescence. Fluorescence dis-tribution maps (Figure 6A) revealed the presence of TVGF-Acp-FITC in various parts of the zebrafish following digestion and catabolism. Yolk sacs of zebrafish treated with TVGF concentrations ranging from 0.05 to 0.30 mg/mL exhibited significant green fluorescence. The head and eyes of zebrafish in 0.20–0.30 mg/mL TVGF group also emitted weak fluorescence. Based on these findings, a concentration of 0.20 mg/mL TVGF was selected for further behavioral analysis. Behavioral experiments demonstrated that TVGF treatment significantly reduced abnormal behavior indicators in zebrafish compared to the IC group (Figure 6B). Specific behavioral changes included decreased moving distance, slower swimming speeds, reduced active time, and increased rest time. These findings suggest that TVGF exhibits beneficial sedative effects by effectively transporting into zebrafish via digestion and catabolism. The observed reduction in abnormal behaviors in zebrafish treated with TVGF highlights its potential as a therapeutic agent for mitigating anxiety-like insomnia.

### 3.7 Transcriptome analysis of zebrafish by TVGF

**FIGURE 7:**
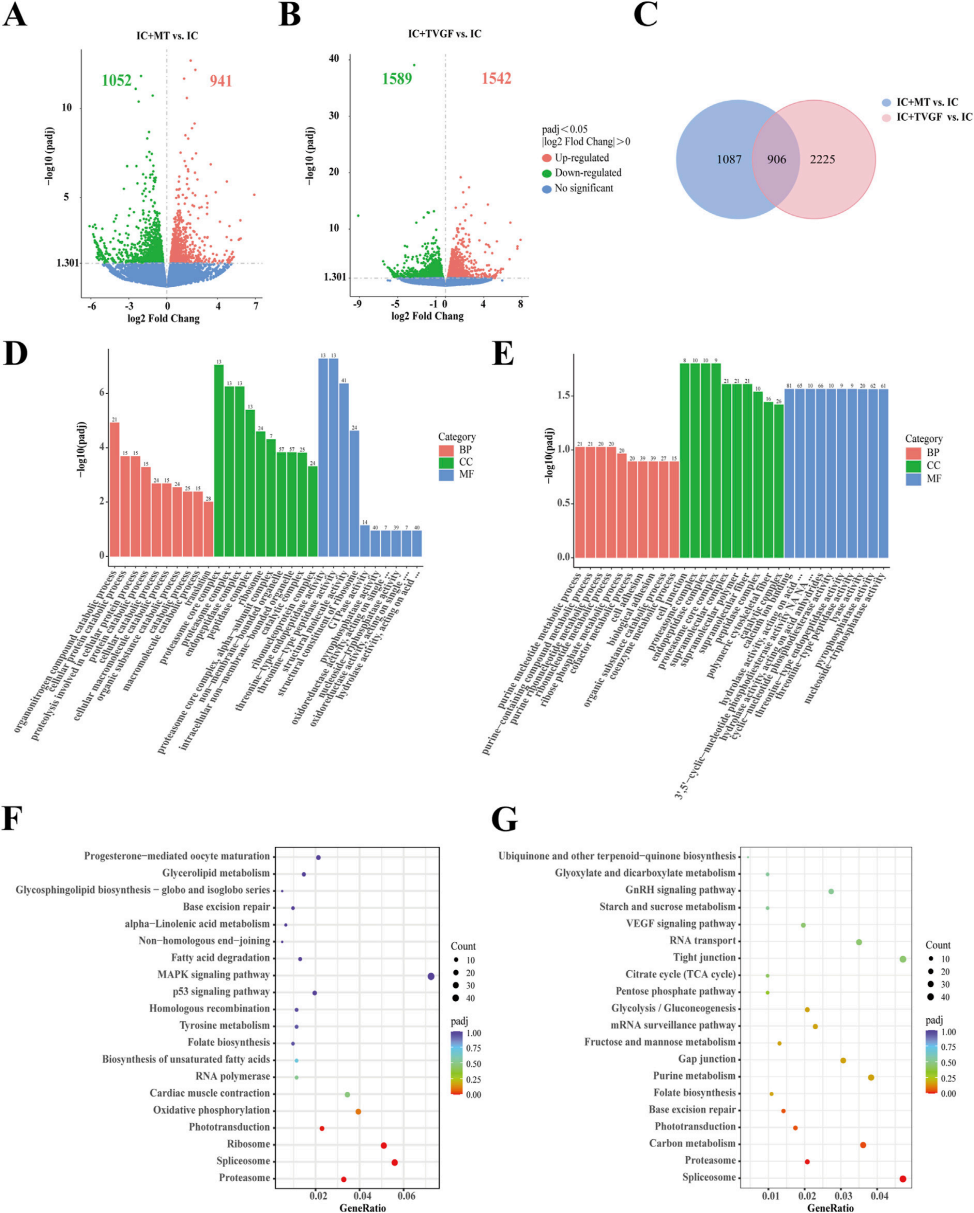
Transcriptome sequencing analysis of TVGF treated zebrafish. Volcano plots depicting differentially expressed genes (DEGs) were generated for both the IC + MT **(A)** and IC + TVGF **(B)** groups, compared to the IC group (n = 20 in each group). **(C)** Venn diagrams illustrated the overlap and unique DEGs identified in the two comparisons: IC + MT vs. IC and IC + TVGF vs. IC. The bar plots of GO enrichment pathways in the IC + MT group **(D)** and IC + TVGF group **(E)** compared with the IC group indicated the top 30 pathways of different genes. The scatter plots of KEGG enrichment pathways in the IC + MT group **(F)** and IC + TVGF group **(G)** compared with the IC group demonstrate the top 20 pathways of different genes.

RNA-seq analysis was performed on zebrafish in the IC, IC + MT and IC + TVGF groups to elucidate the molecular mechanism of synthetic peptides to improve anxiety-like insomnia by analyzing the transcriptome. Detailed quality control analyses are presented in Supplementary Tables S4 and S5. The comparison between the IC + MT and IC groups identified a total of 1993 DEGs (1,052 downregulated and 941 upregulated). Similarly, the IC + TVGF group revealed 3,131 DEGs (1,589 downregulated and 1,542 upregulated) that exhibited differential expression compared to the IC group (Figures 7A,B). A further comparative analysis revealed 906 common DEGs between the two comparisons, while 1,087 DEGs were uniquely regulated by the IC + MT group and 2,225 DEGs were uniquely regulated by the IC + TVGF group (Figure 7C). Similarly, 15 DEGs of greater significance in the two comparison combinations were also screened for analysis (Table 4).

**TABLE 4:** Comparison of the top 15 DEGs in IC + MT and IC + TVGF group compared with IC group.

Gene name	IC + MT vs. IC		IC + TVGF vs. IC	
	log2 FC	Padj	log2 FC	Padj
*per1b*	−2.04	7.90 × 10^−9^	−3.27	1.43 × 10^−35^
*cry1bb*	+1.37	8.31 × 10^−9^	+1.60	5.54 × 10^−16^
*opn1mw1*	+1.99	2.03 × 10^−6^	+2.46	2.24 × 10^−14^
*cry1ba*	+1.58	5.83 × 10^−8^	+1.85	1.28 × 10^−13^
*gnb3b*	+1.69	5.56 × 10^−6^	+2.16	1.29 × 10^−11^
*LOC100332396*			+4.44	1.29 × 10^−11^
*per2*	+1.89	3.23 × 10^−9^	+1.90	3.22 × 10^−11^
*LOC108180286*	−1.82	1.21 × 10^−10^	+1.07	3.49 × 10^−11^
*sik1*			−1.21	1.27 × 10^−10^
*fkbp5*			−1.87	1.86 × 10^−10^
*pnp5a*			−1.75	1.97 × 10^−10^
*arr3b*	+2.19	1.23 × 10^−6^	+2.71	5.40 × 10^−10^
*LOC100536187*			−9.13	5.58 × 10^−10^
*wu:fc34e06*			−2.27	1.05 × 10^−9^
*gnat2*	+2.26	5.34 × 10^−9^	+2.06	3.29 × 10^−9^
*dmtn*	−2.46	2.49 × 10^−8^		
*h3f3d*	−1.11	4.98 × 10^−8^		
*si:dkey-33c14.3*	−2.22	8.09 × 10^−8^		
*crygmx*	−1.40	3.00 × 10^−6^		
*opn1sw1*	+1.47	4.22 × 10^−6^		
*lim2.3*	−1.54	5.56 × 10^−6^		
*mipb*	−1.38	2.91 × 10^−5^		

*Note: log2 FC: log2 Fold Change; −: gene downregulation; +: gene upregulation.

It was observed that several previously reported core circadian genes, including *cry1bb*, *cry1ba* and *per2*, were also upregulated and *per1b* was downregulated in both groups. Furthermore, upregulation of genes associated with visual perception systems such as *opn1mw1*, *gnb3b*, *arr3b* and *gnat2* was observed. Similarly, it was also observed that the TVGF group could individually downregulate the circadian rhythm function gene *sik1* (Jagannath et al., 2013), the core gene *pnp5a* related to purine metabolism (Keller et al., 2022), and the gene *fkbp5* (Ke et al., 2018) related to anxiety disorders mediating the recovery of stress response.

Subsequently, GO and KEGG enrichment analyses were performed. In comparison between the IC and IC + MT groups, the organonitrogen compound catabolic process was the most enriched GO term in biological processes, while the proteasome core complex dominated in cellular composition, and threonine-type endopeptidase activity was mainly enriched in molecular function. In contrast, the IC + TVGF group exhibited a distinct enrichment profile, with the purine nucleotide metabolic process being the most affected biological process. The cell junction was predominantly affected by cell composition, and molecular function was significantly enriched in calcium ion binding (Figures 7D,E) This enrichment in calcium ion binding is crucial for biosynthesis, metabolic regulation, and maintaining calcium homeostasis within mitochondria (Friedman and Nunnari, 2014). KEGG enrichment analysis also identified the most affected the pathways in melatonin and TVGF treatment compared to the IC group. The IC + MT group demonstrated enrichment in the Proteasome, Spliceosome, and Ribosome regulatory pathways, while the IC + TVGF group showed enrichment Spliceosome and Proteasome regulatory pathways (Figures 7F,G).

## 4 Discussion

Insomnia and anxiety show an inextricable bidirectional feedback relationship. While the precise mechanisms behind the sleep-anxiety connection remain unclear, emerging evidence suggests that brain networks may overlap some similar underlying neural circuit mechanisms responsible for insomnia and anxiety (Chellappa and Aeschbach, 2022). MBP1 is a bioactive peptide mixture obtained through enzymatic hydrolysis of mackerel bones, with a molecular weight of less than 1 kDa. MBP1 contains various short peptides, which have demonstrated multiple biological activities in experiments, including antioxidant properties, regulation of neurotransmitter levels, and improvement of sleep disorders. In our study, MBP1 was used as a peptide extract for preliminary functional investigations. The present study induced transient anxiety-related insomnia in zebrafish through 24-h continuous light exposure, disrupting their circadian rhythm. Insomnia was assessed via behavioral changes, neurotransmitter imbalances, and oxidative stress. Behaviorally, zebrafish exhibited increased movement, swimming speed, and active time, with reduced rest. Prolonged light exposure led to higher dopamine (DA) and lower γ-aminobutyric acid (GABA), causing hyperactivity and insomnia. Oxidative stress was reflected by elevated reactive oxygen species (ROS) and malondialdehyde (MDA), along with reduced superoxide dismutase (SOD) and catalase (CAT) activities, linked to brain damage. Notably, different doses of MBP1 reversed these effects and restored DA and GABA concentrations to nearly normal levels, resulting in improved behavioral indicators. Growing evidence suggests that consuming natural substances that regulate neurotransmitter levels can improve sleep quality and regulate the sleep-wake cycle by balancing GABA and serotonin levels (Yan et al., 2019; Si et al., 2020). For instance, plastron of Mauremys mutica peptides improve the disorder of neurotransmitter system and facilitate sleep-promoting in the PCPA-induced insomnia mice (Lv et al., 2021). Jiaotaiwan can improve sleep derived model in rat serum, prefrontal lobe and brain stem of GABA levels, increased the NREM sleep and REM sleep time (SiSi et al., 2022). Therefore, MBP1 can alleviate various behaviors similar to light-induced anxiety by balancing the neurotransmitter systems.

ROS accumulates in neurons during wakefulness, and favorable sleep homeostasis helps support antioxidant defenses to protect against oxidative damage (Bin Heyat et al., 2022). In contrast, anxiety-like insomnia leads to an imbalance in ROS production and endogenous antioxidant defenses, which will negatively affect the immune mechanism and function of the brain system, further damaging neurons (Palagini et al., 2022). MDA is the end product of lipid peroxidation, resulting in the cross-linking of proteins, nucleic acids, and other macromolecules, which can inhibit protein synthesis (Cheng et al., 2020). This is similar to our observation that ROS and MDA levels were significantly elevated in the IC group. Conversely, MBP1 dose-dependent inhibition of ROS production and decreased MDA content demonstrated its potential to regulate oxidative stress. Furthermore, MBP1 scavenged ROS and improved the imbalanced antioxidant system through dose-dependent enhancement of endogenous antioxidant enzyme expression levels (SOD, CAT). Studies have shown that Antarctic krill peptides can effectively enhance SOD activity in serum and reduce the level of MDA in hippocampus of mice (Zheng et al., 2022), while sea cucumber peptide can improve antioxidant capacity and performed well in brain behavior tests (Lu et al., 2022). Thus, we proposed that MBP1 can improve anxiety-like insomnia through enhancing the activity of the antioxidant defense system, in addition to potentially regulating neurotransmitters systems responsible for motor behavior.

Insomnia is a well-defined characteristic in the early detection of many neurodegenerative diseases, and excessive lipid content and failed proteostasis are mutually causative of sleep disorders (Cai et al., 2018; Morrone et al., 2023). A recent study developed a computational chemical microscope incorporating 3D mid-infrared photothermography with fluorescence imaging to achieve 3D visualization of the β-sheet of tau fibril structure and revealed a latent correlation between lipid accumulation and the formation of tau aggregates (Zhao et al., 2023). However, the accumulation of misfolded or unfolded proteins leads to increased ROS generation and thus triggers oxidative stress (Vaccaro et al., 2020). This hypothesis was verified by multi-molecule spectroscopy analysis of lipid and protein characteristic peaks in zebrafish from each group. We discovered that insomnia increased lipid content and decreased protein content, resulting in changes in their secondary structure. Notably, administration of MBP1 at various doses partially improved the dysregulation of lipid accumulation and proteostasis induced by anxiety-like insomnia. As tau aggregation and hyperphosphorylation, neuroinflammation, and oxidative stress are typical pathological hypotheses for the development of various neurodegenerative diseases (Nasb et al., 2024). Protein secondary structure analysis showed that the content of β-sheet protein was higher in the IC group and closer to normal levels in the HP group. It is plausible that MBP1 reduces excessive β-sheet protein accumulation and prevents tau protein aggregation, thus mitigating the adverse effects of lipid accumulation and oxidative stress caused by anxiety-like insomnia.

We mainly focused on transcriptional changes between several hub genes and related signaling pathways in RNA-seq transcription analysis. It was revealed that high doses of MBP1 modulated the expression of genes related to circadian rhythm and energy metabolism in zebrafish, including upregulation of *per2* and downregulation of *sik1* (Jagannath et al., 2013; Vitaterna et al., 2019). Meanwhile, high doses of MBP1 downregulated *opn1mw2* and upregulated *zgc:73075* and *arr3b*, which are known to play crucial roles in visual development (Renninger et al., 2011; Sun et al., 2018). Furthermore, genetic alterations in the circadian rhythm network have been shown to affect visual function in zebrafish, and multiple visual behaviors displayed robust circadian rhythms (Shi et al., 2019), which are consistent with the aforementioned hub genes transcriptional changes. Notably, treatment with 0.3 mg/mL MBP1 altered the transcription of these hub genes with varying patterns and resulted in gradual normal expression. GO enrichment analysis revealed that the HP group was significantly enriched in proteolysis and lipid transport as compared to the IC group, which corresponds to the findings of multi-molecule spectroscopy. This was probably attributed to aberrant expression of circadian genes induced by insomnia, leading to overproduction and overaccumulation of clock proteins. The process involved selectively degrading clock proteins to regulate circadian rhythms (Tamaru and Takamatsu, 2018). Similarly, the main KEGG pathways that were significantly enriched by MBP1 were retinol metabolism, phototransduction, and amino sugar and nucleotide sugar metabolism. Visual perception of zebrafish is generated by opsin secreted from the outer segments of the retinal rod and cone photoreceptors, which capture visible light and initiate a phototransduction cascade, ultimately converting this physical stimulus into biological signals (Ma et al., 2014). Therefore, the relative expression of the light-sensitive opsin gene *opn1mw2* and the cone arrestin gene *arr3b* played a crucial role in the phototransduction pathway. In conclusion, we hypothesize that exogenous MBP1 supplementation may facilitate sleep through the underlying molecular mechanism of chemical composition distribution combined with transcriptional changes in genes related to circadian rhythm and visual function in anxiety-like insomnia zebrafish.

TVGF (Thr-Val-Gly-Phe) is a specific peptide fragment that was further isolated and purified from MBP1. Through mass spectrometry analysis, we identified the sequence of this peptide and further explored its unique physiological functions (Wang et al., 2023). Based on the NovoPro polypeptide net charge calculator (https://www.novopro.cn/tools/calc_peptide_property.html), it was found that TVGF carries a negative charge when pH ≥ 7. Studies have indicated that peptides with neutral or slightly negative charges are more likely to cross the blood-brain barrier (Stalmans et al., 2015). The activity of bioactive peptides influenced by the amino acid composition, number and sequence. Studies have shown that hydrophobic amino acids such as Val and Phe are beneficial to exert their antioxidant activity (Ewert et al., 2022), Previous clinical studies have demonstrated that Gly and Thr can improve sleep quality ((Kawai et al., 2015; Ki and Lim, 2019). Molecular docking verified that TVGF exhibits a good affinity for anxiety-related receptor proteins. Subsequently, behavioral experiments further validated the efficacy of TVGF in alleviating light-induced anxiety-insomnia behavior. Moreover, transcriptome sequencing of zebrafish larvae revealed enrichment of genes involved in circadian rhythm (*per1b*, *cry1bb*, *cry1ba*, *per2*, *sik1*) and visual perception system (*opn1mw1*, *gnb3b*, *arr3b*, *gnat2*), which were significantly associated with critical pathways. These findings suggested that TVGF may similarly promote sleep through transcriptional alterations of circadian clock genes and hub genes in the retinal visual cycle. However, some uniquely regulated genes have also been identified, such as *pnp5a* related to the purine nucleotide metabolic process, and *fkbp5* related to anxiety-related stress response recovery. *fkbp5* is a GR co-chaperone that suppresses GR-mediated modulatory feedback on the hypothalamus-pituitary-adrenal axis in response to emotional stress. Therefore, the elevated level of *fkbp5* results in impaired GR resistance and negative feedback, thus causing anxiety disorders (Ke et al., 2018). The main GO terms enriched in TVGF were the purine nucleotide metabolic process and calcium ion binding, which are essential for biosynthesis, metabolic regulation, and balancing calcium levels in mitochondria (Friedman and Nunnari, 2014). The most significantly enriched KEGG pathways in TVGF were Spliceosome and Proteasome. In the most recent study, 449 genes associated with insomnia were identified by Sherlock integrative analysis, and these genes were significantly overexpressed in the Spliceosome biological pathway (*p* = 1.17 × 10^−4^) (Sun et al., 2020). It was also demonstrated that pharmacological proteasome activation can contribute to the regulation of sleep homeostasis through the degradation of cytoplasmic inclusions of proteins such as Tau and d α-synuclein (Fernández-Cruz et al., 2020). Based on the positive effects of the specific amino acid composition and sequence identified for the TVGF peptide to alleviate anxiety-like insomnia, we can initially confirm that TVGF has great potential for promoting sleep homeostasis.

## 5 Conclusion

Both MBP1 and the novel peptide TVGF demonstrated effectively alleviate light-induced sleep disturbances in zebrafish. Through the observation and analysis of behavioral indicators, neurotransmitter levels, oxidative stress, changes in biological macromolecules and changes in transcription of genes related to circadian rhythm and visual development in zebrafish. MBP1 exhibited a reversal of the adverse effects of light-induced sleep disturbances to varying degrees, approaching the levels observed in the normal group. Further investigation revealed the potential sedative-hypnotic effects of TVGF through molecular docking and genetic analyses. These findings suggest that MBP1 and TVGF alleviate the adverse effects of sleep disturbances mainly by inhibiting oxidative stress and regulating biological rhythms. It is expected to provide new insights and strategies for the development of nutritional supplements for the treatment of insomnia-related diseases.

## Data Availability

The datasets presented in this study can be found in online repositories. The original sequencing data for all transcriptomics samples have been deposited into the NCBI Sequence Read Archive (SRA) public database, with the accession number PRJNA1182301 (https://www.ncbi.nlm.nih.gov/sra/PRJNA1182301).
