# Supplementary material for: Therapeutic potential of mackerel-derived peptides and the synthetic tetrapeptide TVGF for sleep disorders in a light-induced anxiety zebrafish model
Source: Front Pharmacol. 2024 Nov 11;15:1475432. doi: 10.3389/fphar.2024.1475432 (PMC11589825; doi:10.3389/fphar.2024.1475432)
Supplement: Supplementary file 1 [file DataSheet1.docx]

Supplementary Material


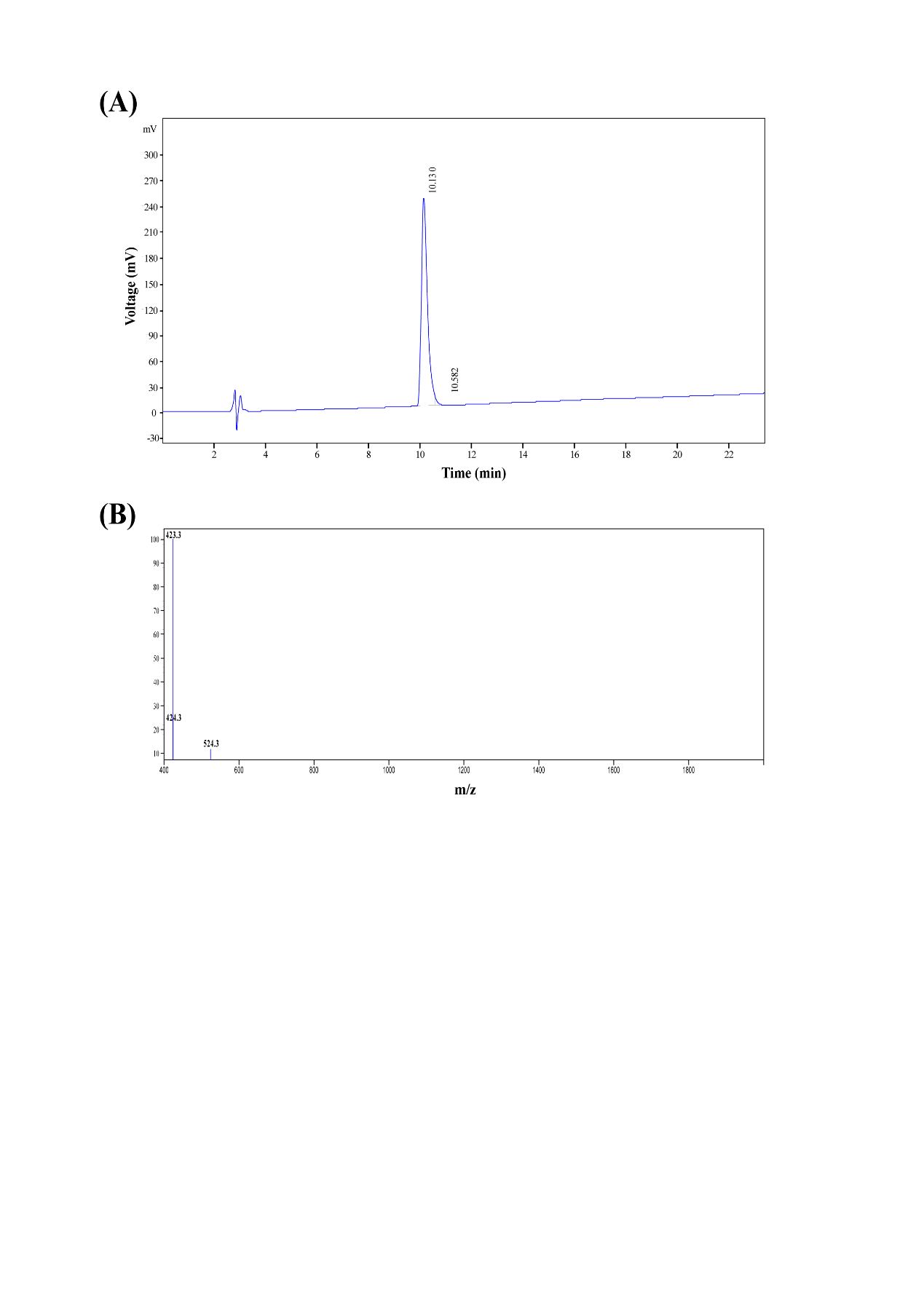


**Supplementary Figure 1.** HPLC and MS (b) analysis of synthetic peptide (TVGF).


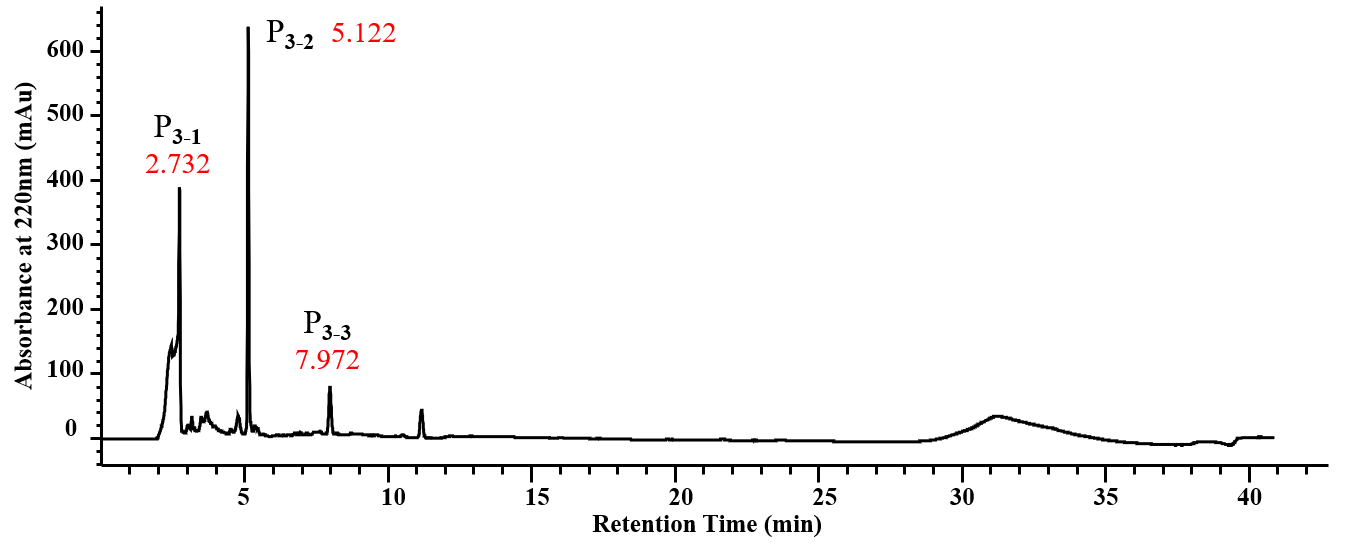


**Supplementary Figure 2.** HPLC analysis of Purified MBP1

The chromatographic conditions are as follows: the injection volume is 20 µL, and the column temperature is set to 30°C. Mobile phase A consists of water with 0.1% formic acid, while mobile phase B is 80% acetonitrile with 0.1% formic acid. The elution gradient is programmed as follows: 0-3 minutes, 2%-6% B; 3-42 minutes, 6%-20% B; 42-47 minutes, 22%-35% B; 47-48 minutes, 35%-100% B; and 48-60 minutes, 100% B.


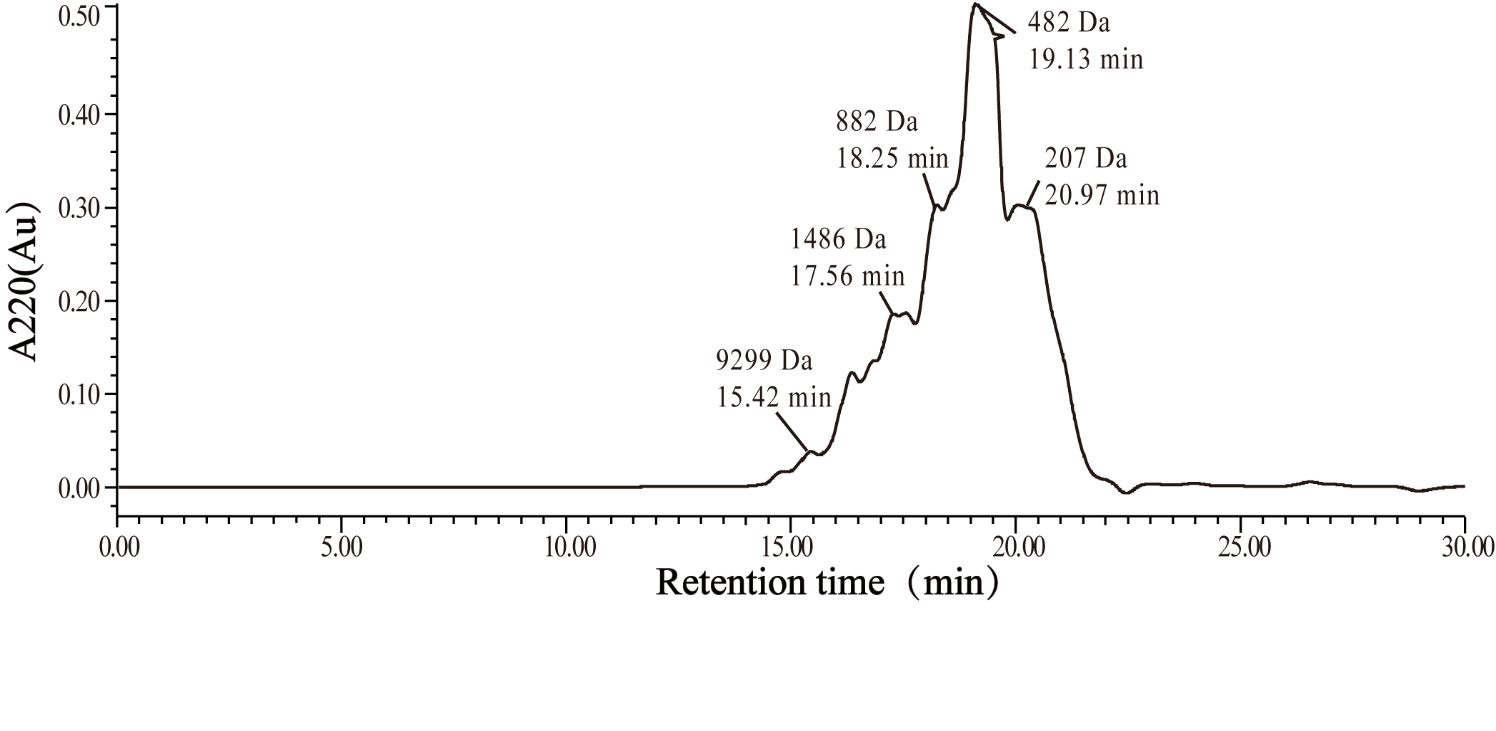


**Supplementary Figure 3. The relative molecular mass distribution of MBP1**

**Supplementary Table 1** Transcriptome sequencing data of zebrafish from different treatment groups (IC, IC+MT, and IC+HP groups).

| Sample | Raw reads | Raw bases | Clean reads | Clean bases | Error rate  (%) | Q20  (%) | Q30  (%) | GC pct  (%) |
| --- | --- | --- | --- | --- | --- | --- | --- | --- |
| IC-1 | 40578096 | 6.09G | 39766994 | 5.97G | 0.03 | 97.34 | 92.75 | 45.26 |
| IC-2 | 42555070 | 6.38G | 41792612 | 6.27G | 0.03 | 97.42 | 92.92 | 44.96 |
| IC+MT-1 | 43499868 | 6.52G | 42651718 | 6.4G | 0.03 | 96.21 | 90.15 | 44.63 |
| IC+MT-2 | 36058272 | 5.41G | 35974152 | 5.4G | 0.03 | 97.52 | 93.25 | 49.37 |
| IC+HP-1 | 47060322 | 7.06G | 46318592 | 6.95G | 0.03 | 96.38 | 90.64 | 45.3 |
| IC+HP-2 | 47122522 | 7.07G | 46492488 | 6.97G | 0.03 | 96.57 | 91.01 | 44.89 |

**Supplementary Table 2** Comparison between clean reads and reference genes in different treatment groups (IC, IC+MT, and IC+HP groups).

| Sample | Total_reads | | | Total_map | Unique_map | Multi_map | Splice_map | Unsplice_map |
| --- | --- | --- | --- | --- | --- | --- | --- | --- |
| IC-1 | | 39766994 | 35845673  (90.14%) | | 34593532  (86.99%) | 1252141  (3.15%) | 13414545  (33.73%) | 21178987  (53.26%) |
| IC-2 | | 41792612 | 37481631  (89.68%) | | 36223985  (86.68%) | 1257646  (3.01%) | 13483902  (32.26%) | 22740083  (54.41%) |
| IC+MT-1 | | 42651718 | 37930605  (88.93%) | | 36716346  (86.08%) | 1214259  (2.85%) | 12969037  (30.41%) | 23747309  (55.68%) |
| IC+MT-2 | | 35974152 | 32448095  (90.2%) | | 31142017  (86.57%) | 1306078  (3.63%) | 15393395  (42.79%) | 15748622  (43.78%) |
| IC+HP-1 | | 46318592 | 41633832  (89.89%) | | 40168724  (86.72%) | 1465108  (3.16%) | 15937903  (34.41%) | 24230821  (52.31%) |
| IC+HP-2 | | 46492488 | 41832618  (89.98%) | | 40372656  (86.84%) | 1459962  (3.14%) | 15587359  (33.53%) | 24785297  (53.31%) |

Protein and peptide sequencing of mackerel samples was performed using a Q-Exactive Plus mass spectrometer, with analysis conducted on an EASY-nLC 1200 system coupled to a C18 microcolumn (75 μm × 15 cm, 3 μm). The mass spectrometric parameters were set as follows: the ion mode used was positive ion mode, with an MS1 scan range of 100-1500 amu and a resolution of 70,000. The MS2 scan range was automatically selected based on the precursor m/z ratio, with a collision energy set at 28% and a resolution of 17,500. The capillary temperature was maintained at 275°C, and the ion source voltage was set to 2300 V. The fragmentation mode employed was HCD.

**Supplementary Table 3** Amino acid sequence of Purified MBP1 identified by LC-MS/MS.

| Peptide | -10 lgP | | Mass | Length | ppm | m/z | RT | Area Sample |
| --- | --- | --- | --- | --- | --- | --- | --- | --- |
| TGVDNPGHPFIK | | 29.11 | 1280.651 | 12 | 5.1 | 427.8932 | 15.54 | 2.00×10^7^ |
| EAGPHGPSGPR | | 28.98 | 1060.505 | 11 | 2.9 | 531.2613 | 7.43 | 3.32×10^5^ |
| NWPTYPQ | | 27.47 | 904.4079 | 7 | 5.3 | 905.42 | 29.46 | 1.25×10^6^ |
| VHHVP | | 26.49 | 587.318 | 5 | 0 | 588.3253 | 42.44 | 0 |
| DGADFAKWR | | 26.19 | 1064.504 | 9 | 4.9 | 533.2618 | 20.02 | 4.97×10^7^ |
| VNIGTIGHVDH | | 25.26 | 1160.594 | 11 | 3 | 581.3059 | 18.22 | 8.19×10^6^ |
| VPLQDVYKIG | | 24.73 | 1130.634 | 10 | 3.3 | 566.3259 | 42.91 | 2.23×10^6^ |
| SYH | | 24.69 | 405.1648 | 3 | 3.7 | 406.1736 | 36.11 | 1.11×10^7^ |
| LDC (+57.02) HTAHIAC (+57.02) | | 24.36 | 1196.507 | 10 | 4.1 | 599.2631 | 10.13 | 8.32×10^5^ |
| GC (+57.02) TSVIC (+57.02) ICK | | 23.87 | 1125.48 | 10 | 1.8 | 563.748 | 9.4 | 1.9×10^6^ |
| HFNDPVH | | 23.83 | 864.3878 | 7 | 5.6 | 433.2036 | 9.07 | 1.86×10^7^ |
| AVDPVYPPGPPAFPK | | 23.69 | 1550.813 | 15 | 2.9 | 776.4161 | 49.32 | 5.83×10^5^ |
| NPLFPPGPPK | | 23.17 | 1062.586 | 10 | 3.2 | 532.3021 | 36.32 | 0 |
| PSGPVGPAGK | | 23.03 | 865.4657 | 10 | 3.8 | 433.7418 | 8.11 | 1.11×10^7^ |
| LYDQHLGK | | 22.1 | 972.5029 | 8 | 2.7 | 487.26 | 9.28 | 1.80×10^6^ |
| LYDQHIGK | | 22.1 | 972.5029 | 8 | 2.7 | 487.26 | 9.28 | 1.80×10^6^ |
| YDQHLGKT | | 21.62 | 960.4665 | 8 | 5.8 | 481.2433 | 7.84 | 9.96×10^5^ |
| YDQHIGKT | | 21.62 | 960.4665 | 8 | 5.8 | 481.2433 | 7.84 | 9.96×10^5^ |
| HDC (+57.02) WPNC (+57.02) T | | 21.39 | 1088.38 | 8 | 4.1 | 545.1997 | 12.05 | 8.94×10^6^ |
| DIVYPPGPPSNPR | | 21.14 | 1407.715 | 13 | 3.8 | 704.8672 | 29.34 | 1.98×10^7^ |
| GFAGDDAPR | | 21.04 | 904.4039 | 9 | 3.4 | 453.2108 | 9.53 | 2.77×10^7^ |
| VSFPY | | 20.81 | 611.2955 | 5 | 2 | 612.304 | 34.44 | 1.50×10^7^ |
| YGNPWEK | | 20.56 | 892.4079 | 7 | 2.6 | 447.2124 | 13.46 | 1.55×10^8^ |
| HERDPTQI | | 20.22 | 994.4832 | 8 | 5.6 | 498.2516 | 8.6 | 2.06×10^6^ |
| IDK | | 20.16 | 374.2165 | 3 | 3.6 | 375.2251 | 8.47 | 6.09×10^7^ |
| LDK | | 20.16 | 374.2165 | 3 | 3.6 | 375.2251 | 8.47 | 6.09×10^7^ |
| AGFAGDDAPR | | 20.08 | 975.441 | 10 | 2.5 | 488.729 | 10.32 | 1.16×10^7^ |
| **TVGF** | | **20.03** | **422.2165** | **4** | **2.1** | **423.2247** | **20.87** | **1.31×10^8^** |

**Supplementary Table 4** Transcriptome sequencing data of zebrafish from different treatment groups (IC, IC+MT, and IC+TVGF groups).

| Sample | Raw Reads | Raw bases | Clean reads | clean bases | Error rate  (%) | Q20  (%) | Q30  (%) | GC pct  (%) |
| --- | --- | --- | --- | --- | --- | --- | --- | --- |
| IC-1 | 42122530 | 6.32G | 40116770 | 6.02G | 0.02 | 97.08 | 94.26 | 43.78 |
| IC-2 | 45194900 | 6.78G | 42512898 | 6.38G | 0.02 | 97.97 | 94.36 | 44.01 |
| IC-3 | 45435116 | 6.82G | 42057022 | 6.31G | 0.03 | 96.77 | 91.56 | 44.37 |
| IC+MT-1 | 42549722 | 6.38G | 40956028 | 6.14G | 0.03 | 97.92 | 94.14 | 43.52 |
| IC+MT-2 | 44901536 | 6.74G | 42165598 | 6.32G | 0.03 | 97.86 | 94.14 | 44.3 |
| IC+MT-3 | 44275970 | 6.64G | 41996394 | 6.3G | 0.03 | 97.83 | 94.03 | 43.88 |
| IC+TVGF-1 | 44583762 | 6.69G | 42511374 | 6.38G | 0.02 | 97.97 | 94.37 | 44.53 |
| IC+TVGF-2 | 42185776 | 6.33G | 40427474 | 6.06G | 0.03 | 97.88 | 94.05 | 43.64 |
| IC+TVGF-3 | 43649508 | 6.55G | 41870864 | 6.28G | 0.03 | 97.79 | 94.03 | 44.39 |

**Supplementary Table 5** Comparison between clean reads and reference genes in different treatment groups (IC, IC+MT, and IC+TVGF groups).

| Sample | Total_reads | Total_map | Unique_map | Multi_map | Splice_map | Unsplice_map |
| --- | --- | --- | --- | --- | --- | --- |
| IC-1 | 40116770 | 36508981  (91.01%) | 31397088  (78.26%) | 5111893  (12.74%) | 8483249  (21.15%) | 22913839  (57.12%) |
| IC-2 | 42512898 | 38591477  (90.78%) | 32929190  (77.46%) | 5662287  (13.32%) | 9420765  (22.16%) | 23508425  (55.3%) |
| IC-3 | 42057022 | 37236170  (88.54%) | 31928163  (75.92%) | 5309007  (12.62%) | 9420746  (22.4%) | 22507417  (53.52%) |
| IC+MT-1 | 40956028 | 37103956  (90.59%) | 31930958  (77.96%) | 5172998  (12.63%) | 8520220  (20.8%) | 23410738  (57.16%) |
| IC+MT-2 | 42165598 | 37808838  (89.67%) | 32342871  (76.7%) | 5465967  (12.96%) | 9115767  (21.62%) | 23227104  (55.09%) |
| IC+MT-3 | 41996394 | 37758445  (89.91%) | 32436381  (77.24%) | 5322064  (12.67%) | 9061089  (21.58%) | 23375292  (55.66%) |
| IC+TVGF-1 | 42511374 | 38454470  (90.46%) | 32805043  (77.17%) | 5649427  (13.29%) | 9520778  (22.4%) | 23284265  (54.77%) |
| IC+TVGF-2 | 40427474 | 36589982  (90.51%) | 31287785  (77.39%) | 5302197  (13.12%) | 8166791  (20.2%) | 23120994  (57.19%) |
| IC+TVGF-3 | 41870864 | 37071751  (88.54%) | 31711277  (75.74%) | 5360474  (12.8%) | 8961158  (21.4%) | 22750119  (54.33%) |

**Supplementary Table 6** Amino acid composition of MBP1

| Amino acid | g/100g |
| --- | --- |
| Aspartic acid (Asp) | 0.72 ± 0.01 |
| Threonine (Thr) | 0.43 ± 0.00 |
| Serine (Ser) | 0.45 ± 0.01 |
| Glutamic acid (Glu) | 0.96 ± 0.01 |
| Glycine (Gly) | 0.72 ± 0.01 |
| Alanine (Ala) | 0.87 ± 0.01 |
| Valine (Val) | 0.83 ± 0.03 |
| Methionine (Met) | 0.087 ± 0.01 |
| Isoleucine (lle) | 0.22 ± 0.01 |
| Leucine (Leu) | 0.65 ± 0.03 |
| Tyrosine (Tyr) | 0.12 ± 0.01 |
| Phenylalanine (Phe) | 2.18 ± 0.20 |
| Lysine (Lys) | 1.00 ± 0.21 |
| Histidine (His) | 0.26 ± 0.09 |
| Arginine (Arg) | 0.97 ± 0.03 |
| Proline (pro) | 0.32 ± 0.20 |
| Total Amino Acids(TAA) | 10.79 |

Based on the previous research data of the research group.
